# SgRVE6, a LHY-CCA1-Like Transcription Factor From Fine-Stem Stylo, Upregulates NB-LRR Gene Expression and Enhances Cold Tolerance in Tobacco

**DOI:** 10.3389/fpls.2020.01276

**Published:** 2020-08-19

**Authors:** Shu Chen, Huai-An Huang, Jian-Hui Chen, Cheng-Cheng Fu, Peng-Lin Zhan, Shan-Wen Ke, Xiang-Qian Zhang, Tian-Xiu Zhong, Xin-Ming Xie

**Affiliations:** ^1^Department of Grassland Science, College of Forestry and Landscape Architecture, South China Agricultural University, Guangzhou, China; ^2^Guangdong Engineering Research Center for Grassland Science, Guangzhou, China; ^3^Gansu Engineering Laboratory of Applied Mycology, Hexi University, Zhangye, China

**Keywords:** RVE transcription factor, cold tolerance, circadian clock, fine-stem stylo, nucleotide binding domain leucine-rich repeats

## Abstract

*Stylosanthes* species are economically important tropical and subtropical forage legumes which are generally vulnerable to chilling and frost. Fine-stem stylo (*S. guianensis* var. *intermedia*) has the most superior cold tolerance among all stylo species. A *REVEILLE* (*RVE*) gene, *SgRVE6*, was cloned from fine-stem stylo. Bioinformatic analysis suggests that *SgRVE6* encodes a transcription factor of 292 amino acid residues, which belongs to the LATE ELONGATED HYPOCOTYL/CIRCADIAN CLOCK ASSOCIATED 1-LIKE (LCL) subgroup of RVE family and contains a SHAQKYF-class MYB domain and a LCL domain. *SgRVE6* is universally expressed in root, stem and leaf tissues of fine-stem stylo and is rapidly up-regulated in all tested tissues under cold stress. Over-expressing *SgRVE6* affects expression of 21 circadian clock genes, up-regulates expression of 6 nucleotide binding domain leucine-rich repeats (NB-LRR) encoding genes associated with tobacco cold tolerance, improves physiological responses to low temperature, and endows the transgenic tobaccos with higher tolerance to cold stress. This is the first time a study investigates the biological function of RVE6 in cold responses of plant species.

## Introduction

Stylo (*Stylosanthes guianensis*) is an economically important forage and pasture legume widely cultivated throughout tropical, subtropical and temperate regions of Asia, Africa, Americas and Australia (Noble et al., 2000). In China, stylo is mainly used as a cover crop in plantations, which controls weed growth and improves soil organic matter, and later can be harvested as fresh feed for animals or cut and dried for leaf meal production (Grigg et al., 2000). Stylo is well adapted to drought and soil infertility, but very vulnerable to low temperature and frost, which is the major factor limiting its growth and survival in subtropical and temperate regions (Zhou et al., 2005; Bao et al., 2016). Fine-stem stylo (*S. guianensis* var. *intermedia*), also called Oxley, is first collected by William Hartley in Paraguay and the Argentine (Humphrey, 1980), and has been an effective and persistent legume in the subtropics of Australia and Zimbabwe (Chakraborty, 2004). Fine-stem stylo is well adapted to drought, infertility and low-grazing (persisting after being grazed close to ground for months), but most remarkably fine-stem stylo has the best cold and frost tolerance among all stylo varieties and cultivars. Unveiling the molecular mechanisms underlying the superior cold tolerance of fine-stem stylo will greatly contribute to breeding cold and frost tolerant stylos.

A common strategy to study stress-responsive mechanisms in plants is to identify stress-responsive genes and to verify their functions in stress responses or tolerance, but till today only one stylo gene, *9-cis-epoxycarotenoid dioxygenase* (*SgNCED1*), has been verified to be associated with chilling tolerance by molecular methods of gene function characterization (Yang and Guo, 2007; Bao et al., 2016). Considering the poor genetic information sources in stylo and the outstanding cold and frost tolerance of fine-stem stylo, whole-transcriptome expression profiles of fine-stem stylos under normal (25°C) and cold (4°C) condition were compared (NCBI Bioproject PRJNA277095). The results revealed that a gene encoding a REVEILLE (RVE) transcription factor was remarkably over-expressed under cold condition and therefore selected as the candidate gene for the study of cold tolerance in fine-stem stylo. Bioinformatic analysis revealed that the candidate gene encoded a transcription factor homologous to RVE6, a member of RVE transcription factor family, and therefore the candidate gene was named as *SgRVE6*. The members of the RVE family have been shown to function in the classic circadian clock loop in *Arabidopsis thaliana* (Zhang et al., 2007; Farinas and Mas, 2011; Rawat et al., 2011; Hsu et al., 2013; Fogelmark and Troein, 2014), and consists of CIRCADIAN CLOCK ASSOCIATED 1 (CCA1), LATE ELONGATED HYPOCOTYL (LHY), and nine RVEs (Farinas and Mas, 2011; Rawat et al., 2011). A few studies showed that sensitivity to abiotic stresses was altered in *CCA1/LHY/RVE* mutants of *A. thaliana*. For example, a *cca1/lhy* mutant exhibited greater sensitivity to salt, osmotic and heat stress (Cao et al., 2005; Kant et al., 2008), while a *rve1* mutant showed greater tolerance to low temperature (Meissner et al., 2013), indicating that these RVE members play important roles in cold responses. In fact, not just the RVE members, several other clock components have been proven to regulate cold responses in plants, e.g., PRR9/7/5/1 are repressors of C-REPEAT BINDING FACTOR-COLD REGULATED (CBF-COR) cold responsive pathway and negatively regulate cold tolerance in *Arabidopsis* (Nakamichi et al., 2009; Nakamichi et al., 2012), and GIGANTEA (GI) confers freezing tolerance to *Arabidopsis* in a CBF independent manner (Cao et al., 2005). Transcriptomic analysis revealed that many cold responsive genes were under the control of circadian clock, and the rhythmic expression of cold-responsive genes established the basis of rhythmic variations in cold tolerance. In *Arabidopsis*, 41% of cold regulated genes were found to be rhythmically expressed in constant light (Covington et al., 2008). The majority of cold-inducible genes peaked in the afternoon, a few hours before temperature starts to drop at night, and the cold-repressed genes peaked around dawn when temperature starts to rise, which demonstrated that the rhythmic expression of cold-responsive genes functions to predict and adapt to diurnal temperature variation (Dong et al., 2011). Considering our RNA-seq results and the regulatory role of circadian clock in abiotic-stress tolerance, we speculated that SgRVE6 might participate in the regulation of cold responses and contribute to the superior cold tolerance of fine-stem stylo.

In this paper, the expression pattern of *SgRVE6* under cold condition was determined in leaf, stem and root tissues of fine-stem stylo. Transgenic tobacco lines over-expressing *SgRVE6* (*SgRVE6*-OEs) were generated. Phenotypical appearance and physiological variations were determined in the transgenic tobaccos under cold condition. The results indicated that *SgRVE6* was rapidly induced by low temperature and intrigue a series of physiological changes to counteract cold stress, and accordingly enhance the cold tolerance of tobacco plants. Whole-transcriptome expression profiling was performed on wild-type and two *SgRVE6*-OE tobacco lines using RNA-seq analysis. Co-analysis with two previous RNA-seq studies on cold sensitive and tolerant tobaccos under cold stress revealed that nucleotide binding domain leucine-rich repeats (NB-LRR) genes were significantly induced by SgRVE6 and might be responsible for the improved cold tolerance in tobacco. Although a few studies have demonstrated the association between abiotic stress tolerance and other members of the RVE family, such as CCA1, LHY, and RVE1, this is the first study that investigates the role of RVE6 in regulation of cold responses in plant species.

## Materials and Methods

### Gene Cloning and Sequence Analysis

Total RNA was extracted from the fresh leaf of the fine-stem stylo plants using TransZol Plant Kit (TransGen Biotech, China). The first cDNA was synthesized using TaKaRa PrimeScript II 1st Strand cDNA Synthesis Kit (Takara Biotechnology, Japan). The full-length coding DNA sequence (CDS) of *SgRVE6* was amplified using the primer pair *SgRVE6*-F and *SgRVE6*-R (**Supplementary Table 1**). The primers were designed based on *SgRVE6* transcript sequence from our previous transcriptome assembly. The amino acid sequences of SgRVE6 was aligned with 14 leguminous RVE6s (**Supplementary Table 2**) and the 11 members of the RVE family in *A. thaliana* (**Supplementary Table 3**) using T-Coffee. Phylogenetic trees were constructed from the alignment by Unipro UGENE1.27 using MrBayes method. The physicochemical property of SgRVE6 was analyzed by ProtParam (http://web.expasy.org/protparam/).

### Expression Analysis Under Cold Treatment

Ten fine-stem stylo plants were vegetatively propagated by cuttings. Fresh shoots of 10 cm were cut from a single fine-stem stylo plant and cultured in water for rooting. After one week, rooted shoots were planted in pots (10 cm in height and 12 cm in diameter) containing soil mixtures of Jiffy^®^ substrate and vermiculite (3:1) and cultured in a growth chamber at 28°C and 70% humidity under a 16/8-h (light/dark) photoperiod for four weeks. Plants of uniform growth were selected for cold treatment at 4°C and 70% humidity under a 16/8-h (light/dark) photoperiod. Root, leaf and stem tissues were harvested at 0, 2, 6, and 12 h after cold treatment. Total RNA was extracted from nitrogen-frozen tissues using TransZol Plant Kit (TransGen Biotech, China). First-strand cDNA was synthesized using TaKaRa PrimeScript II 1st Strand cDNA Synthesis Kit (Takara Biotechnology, Japan). The synthesized cDNA was used as template for Real-time PCR in a CFX-96 Real-time System (BioRad, USA) with the specific primer pair, *SgRVE6*-F1 and *SgRVE6*-R1 (**Supplementary Table 1**). Real-time PCR was conducted using SYBR Premix Ex Taq II kit (Takara Biotechnology, Japan). The Real-time PCR reaction solution and procedure followed the manufacturer’s instruction. The results were analyzed by Bio-Rad CFX Manager Software 1.6.

### Tobacco Transformation

An efficient regeneration system of stylo has been established in our lab, but it is still very difficult to obtain a transgenic stylo plantlet through *Agrobacterium*-mediated transformation due to the low transformation efficiency and the long periodicity of callus induction and regeneration. Thus, a model plant tobacco was used as the model plant to verify the function of SgRVE6 in this study. *SgRVE6* CDS was amplified using the primer pair *SgRVE6*-PBA-F and *SgRVE6*-PBA-R (**Supplementary Table 1**), and fused into an over-expression vector pBA002 carrying spectinomycin resistance gene and basta resistance (*bar*) gene. The recombinant plasmid, pBA002-*SgRVE6*, was transformed into *Agrobacterium tumefaciens* strain EHA105. Tobacco leaf disks were infected with the transformed EHA105 strains when bacterial concentration reached 0.5 to 0.6 (OD_600_). The infected leaf explants were placed on co-cultivation MS medium with 0.1 mg L^−1^ NAA and 0.5 mg L^−1^ 6-BA for 3 days. After co-cultivation, the leaf disks were placed on selection MS medium with 0.5 mg L^−1^ 6-BA, 0.1 mg L^−1^ NAA, cefotaxime 500 mg L^−1^ and basta 5 mg L^−1^. Shoots of 2 to 3 cm were transferred onto rooting 1/2 MS medium with 0.2 mg L^−1^ NAA and 100 mg L^−1^ timetin. Plantlets with well-developed roots were transplanted to Jiffy soil (Jiffy Products International AS, Norway) and cultivated at 25°C under a 16/8-h (light/dark) photoperiod.

### Verification and Molecular Analysis of Transgenic Plants

Fifteen days after transplanted to soil, transgenic and wild-type (WT) tobacco leaf surface was painted with 0.2% (W/V) basta. One week after the basta treatment, plantlets with no leaf injury were selected for further investigation. DNA was isolated from fresh leaves using Plant Genomic DNA Kit (TianGen Biotech, China) and amplified with two primer pairs, *bar*-F and *bar*-R, *SgRVE6*-F1 and *SgRVE6*-R1 (**Supplementary Table 1**). Total RNA was isolated from fresh leaves using RNAprep Pure Plant Kit (TianGen Biotech, China). The first cDNA was synthesized using TaKaRa PrimeScript II 1st Strand cDNA Synthesis Kit (Takara Biotechnology, Japan) and used for PCR amplification with primer pair, *SgRVE6*-F2 and *SgRVE6*-R2 (**Supplementary Table 1**). The WT plants served as a negative control and pBA002-*SgRVE6* plasmid carrying *bar* gene and *SgRVE6* as a positive control. The transgenic and WT tobacco plants were cultivated at 25 °C and 70% humidity under a 16/8-h (light/dark) photoperiod with a photon flux density (PFD) of 60 μmol m^−2^ s^−1^.

### Cold Treatment and Physiological Determination

One month after transferred to soil, transgenic and WT tobacco plants of uniform growth were selected and exposed to cold treatment at 4°C and 70% humidity under a 16/8-h (light/dark) photoperiod with a photon flux density (PFD) of 60 μmol m^−2^ s^−1^. Fresh leaves were harvested after cold treatments of 0, 2, 6, 12, 24, and 48 h, and used for determination of proline content (Zhang et al., 2012), MDA content (Cui and Wang, 2006), soluble sugar content (Zhang et al., 2012), relative electrolyte leakage and soluble protein content (Zhang et al., 2012). Three replicates were conducted for each measurement.

### RNA-Seq Analysis of SgRVE6-OE Tobacco

Total RNA was isolated from the third leaves of wild-type and two *SgRVE6* over-expressing tobacco lines (*SgRVE6*-OE3 and *SgRVE6*-OE5, T2 generation) using RNAprep Pure Plant Kit (TianGen Biotech, China), respectively. The tobacco plants were in eight-leaf stage and cultured at 25°C under a 16/8-h (light/dark) photoperiod. The RNA was electrophoresed on 1% agarose gels for monitoring RNA degradation and contamination. RNA purity, concentration, and integrity were further assessed using Qubit2.0 (Thermo Scientific, USA) and Agilent 2100 (Agilent Technologies, USA). Nine RNA-Seq libraries (3 tobacco lines × 3 independent biological replicates) were prepared using the NEBNext^®^ Ultra™ Directional RNA Library Prep Kit for Illumina^®^ (NEB, USA), following manufacturer’s instructions. RNA-seq libraries were sequenced using Illumina HiSeq X-ten platform at Biomarker Technologies Corporation, Beijing, China.

Raw sequencing reads were processed with a perl script to remove adapters and primers, and discard reads containing over 5% ambiguous base N or over 50% low quality bases (quality score < 10). The Clean reads were mapped to the reference genome of *Nicotiana_tabacum* cv. K326 (ftp://ftp.solgenomics.net/genomes/Nicotiana_tabacum/edwards_et_al_2017/assembly/Nitab-v4.5_genome_Scf_Edwards2017.fasta.gz) using HISAT2 (Edwards et al., 2017; Kim et al., 2019). Gene expression was quantified using StringTie v2.0.4 (Pertea et al., 2016). Pairwise gene expression comparisons were performed between WT and *SgRVE6*-OE3, WT and *SgRVE6*-OE5 using DESeq2 v1.26.0 (Love et al., 2014). Genes with FDR ≤ 0.05 and absolute value of log_2_ fold change (log_2_FC) ≥ 1 were identified as differentially expressed genes (DEGs). GO and KEGG enrichment analysis of DEGs was performed using the clusterProfiler R package (Yu et al., 2012). The clean reads were deposited to Sequence Read Archive (SRA) database at National Center for Biotechnology Information (NCBI) under BioProject PRJNA608154.

### RNA-Seq Analysis of Tobaccos Under Low Temperature

To investigate the SgRVE6-induced genes in response to low temperature, raw Illumina sequencing reads were downloaded from two BioProjects, PRJNA368913 and PRJNA287250. PRJNA368913 contains RNA-seq data for tobacco CB-1 (cold-sensitive) and K326 (cold-tolerance) before and after 24-h (h) cold treatment (4°C), and two replicates were available for each tobacco line for each treatment (Jin et al., 2017). RNA-seq analysis followed the same pipeline used in the RNA-seq analysis of *SgRVE6*-OE tobacco. PRJNA287250 contains RNA-seq data of tobacco Taiyan8 (cold-sensitive) and NC567 (cold-tolerance) under cold stress (0, 4, 24 and 48 h, 6°C), and no replicate was available for each bio-sample (Hu et al., 2016). Thus, GFOLD, a bioinformatic tool designed to deal with RNA-seq experiments without replicates, was used to identify DEGs. Genes with GFOLD > 0 and log_2_FC ≥ 1 were identified as up-regulated genes, and genes with GFOLD < 0 and log_2_FC ≤ −1 were identified as down-regulated genes.

### Statistical Analysis

All physiological and Real-time PCR data was analyzed using the IBM SPSS Statistics 23.0 software. Significance of differences between samples or treatments were evaluated by Duncan’s multiple range test.

## Results

### SgRVE6 Is a New Member to RVE Family

**Figure 1:**
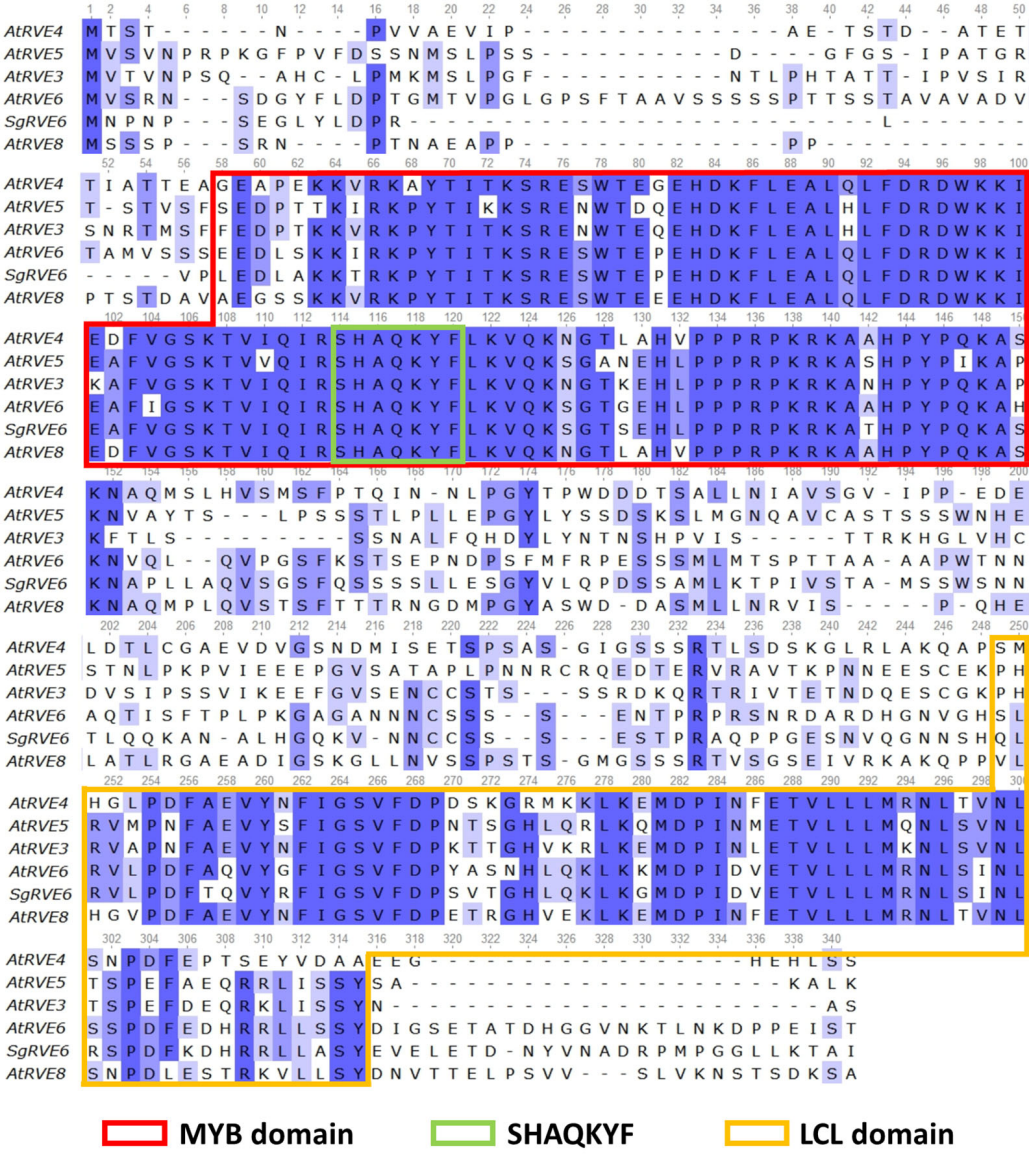
Amino acid sequence alignment of the *Arabidopsis thaliana* LHY/CCA1-LIKE (LCL) subfamily and SgRVE6. The alignment was performed using the T-Coffee multiple sequence alignment. The blue backgrounds correspond to percent identity of the multiple alignment. The boxes delimit the MYB domain (red), the SHAQKYF motif (green) and the LCL domain (yellow).

**Figure 2:**
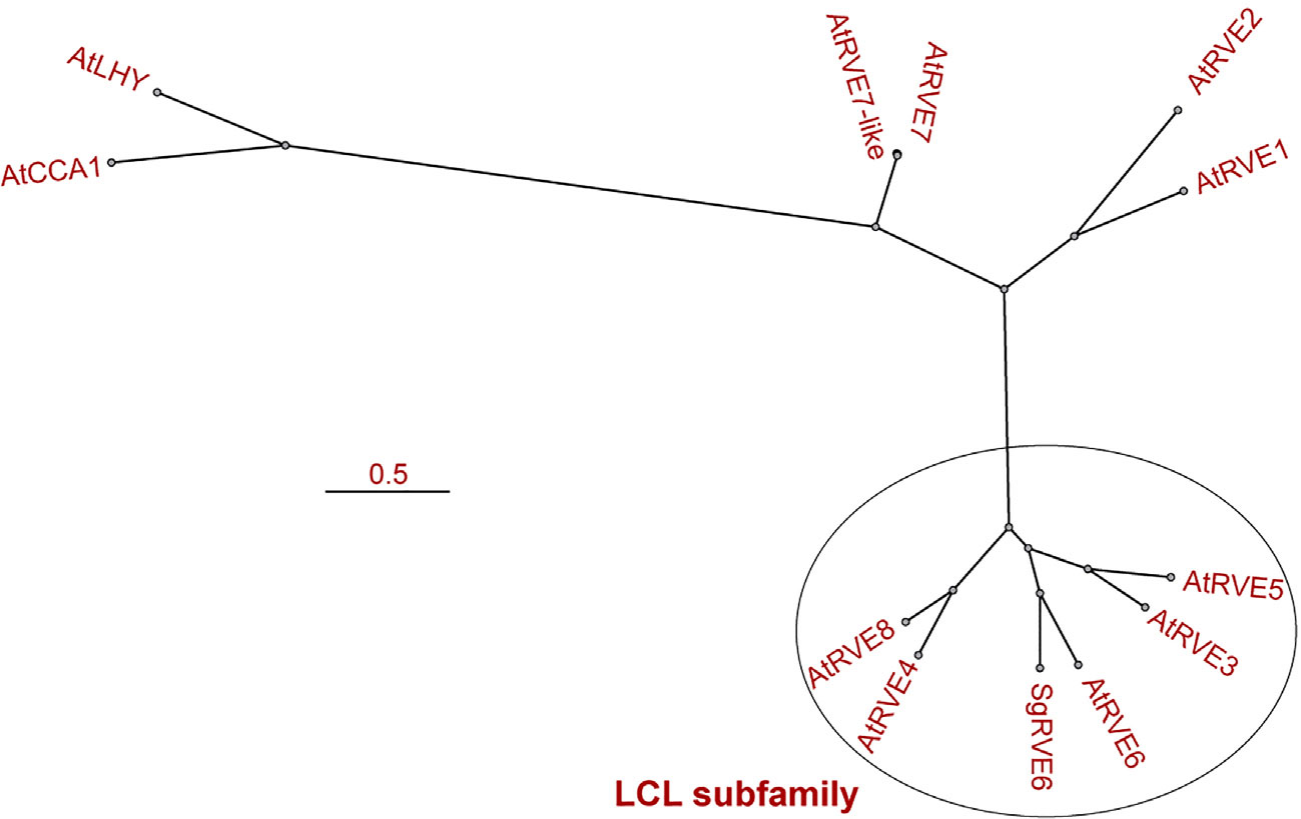
Phylogenetic analysis of the eleven members of the *Arabidopsis thaliana* RVE family and SgRVE6. T-Coffee was used for the multiple alignment and MrBayes for the tree construction. The oval delimits the members of LHY/CCA1-LIKE (LCL) subfamily. The scale bar represents the number of nucleotide substitutions per site.

The *SgRVE6* CDS was obtained from fine-stem stylos by PCR and Sanger sequencing (GenBank accession number: MF596169). ProtParam analysis showed that *SgRVE6* encodes a polypeptide of 292 amino acid residues with a predicted molecular mass of 32.71 kD. High conservation level was observed in the alignment of SgRVE6 and RVE6 protein sequences of other ten legume species (**Supplementary Table 2**). Percent identities between SgRVE6 and other leguminous RVE6 proteins ranged from 73% to 92%. Among the ten leguminous RVE6 proteins, AiRVE6 (*Arachis ipaensis*) and AdRVE6 (*Arachis duranensis*) were closest to SgRVE6, showing percent identities of 92% and 91%, respectively (**Supplementary Figure 1**). The phylogenetic analysis also clustered SgRVE6 with AiRVE6 and AdRVE6 among the ten leguminous RVE6s (**Supplementary Figure 2**). To further clarify the homology of SgRVE6 to RVE family, SgRVE6 was aligned with the eleven members of the *A. thaliana* RVE family. The alignment of SgRVE6 and the five members of the LCL subfamily showed that SgRVE6 contains a SHAQKYF-class MYB domain and a LCL domain (**Figure 1**). The phylogenetic analysis showed that SgRVE6 was closest to AtRVE6 and clustered into the LHY/CCA1-LIKE (LCL) subgroup of the RVE family (**Figure 2**).

### SgRVE6 Is a Cold Responsive Transcription Factor

**Figure 3:**
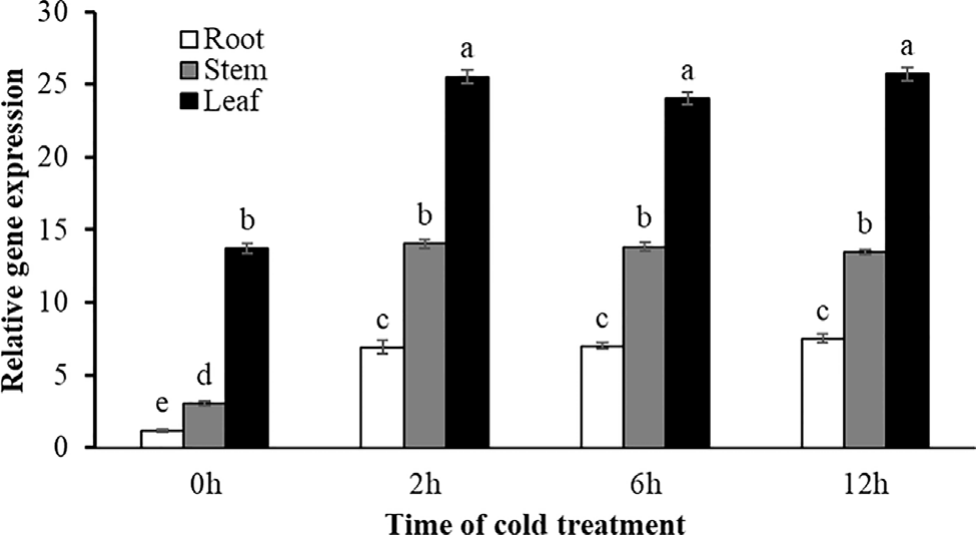
Expression variation of *SgRVE6* in stylo root, stem and leaf under normal and cold condition. Root, stem and leaf tissues of stylo seedlings were collected 0 (control), 2, 6, and 12 h after cold treatment and used for qRT-PCR. Relative expression was compared to actin gene and presented as means ± SD of three sample replicates. The same letter above the columns indicated no significant difference at *P*<0.01 with the corresponding control of each tissue type.

Expression variations of *SgRVE6* under cold condition (4°C) were determined in root, stem and leaf tissues of fine-stem stylo plants using Real-time PCR. The results showed that *SgRVE6* was universally expressed in the root, leaf and stem tissues of fine-stem stylo. No matter under normal or cold condition, the *SgRVE6* expression was highest in leaf and lowest in root. Under normal condition, the *SgRVE6* expression in leaf was 4.5 times of that in stem and 11.6 times of that in root, while under cold condition, the *SgRVE6* expression in leaf was 1.7 to 1.9 times of that in stem and 3.4 to 3.7 times of that in root. Although the *SgRVE6* expression level was different among root, stem, and leaf, a similar pattern of expression variation was observed in the three different tissues under cold condition. When fine-stem stylo seedlings were subjected to cold stress, the *SgRVE6* expressions in leaf, stem and root tissues all increased rapidly and peaked after 2-h cold treatment. The expressions remained at the increased level as the cold treatment continued (**Figure 3**).

### Generation of SgRVE6 Overexpressing Tobaccos

The constructed over-expression vector pBA002-*SgRVE6* was transformed into tobaccos by *Agrobactium tumefaciens*. Seven putative transgenic plants, *SgRVE6*-OE1-7, were obtained from selection medium, and *SgRVE6*-OE3-7 were verified as *SgRVE6* over-expressing transgenic plants by amplification of *bar* and *SgRVE6* genes and foliar application of basta (**Supplementary Figure 4**). T2 generation of *SgRVE6*-OE3, 4, and 5 were used to determine cold tolerance, and T2 generation of *SgRVE6*-OE3 and 5 were used for RNA-seq analysis.

### Enhanced Responses to Cold Stress in SgRVE6-OE Tobacco

**Figure 4:**
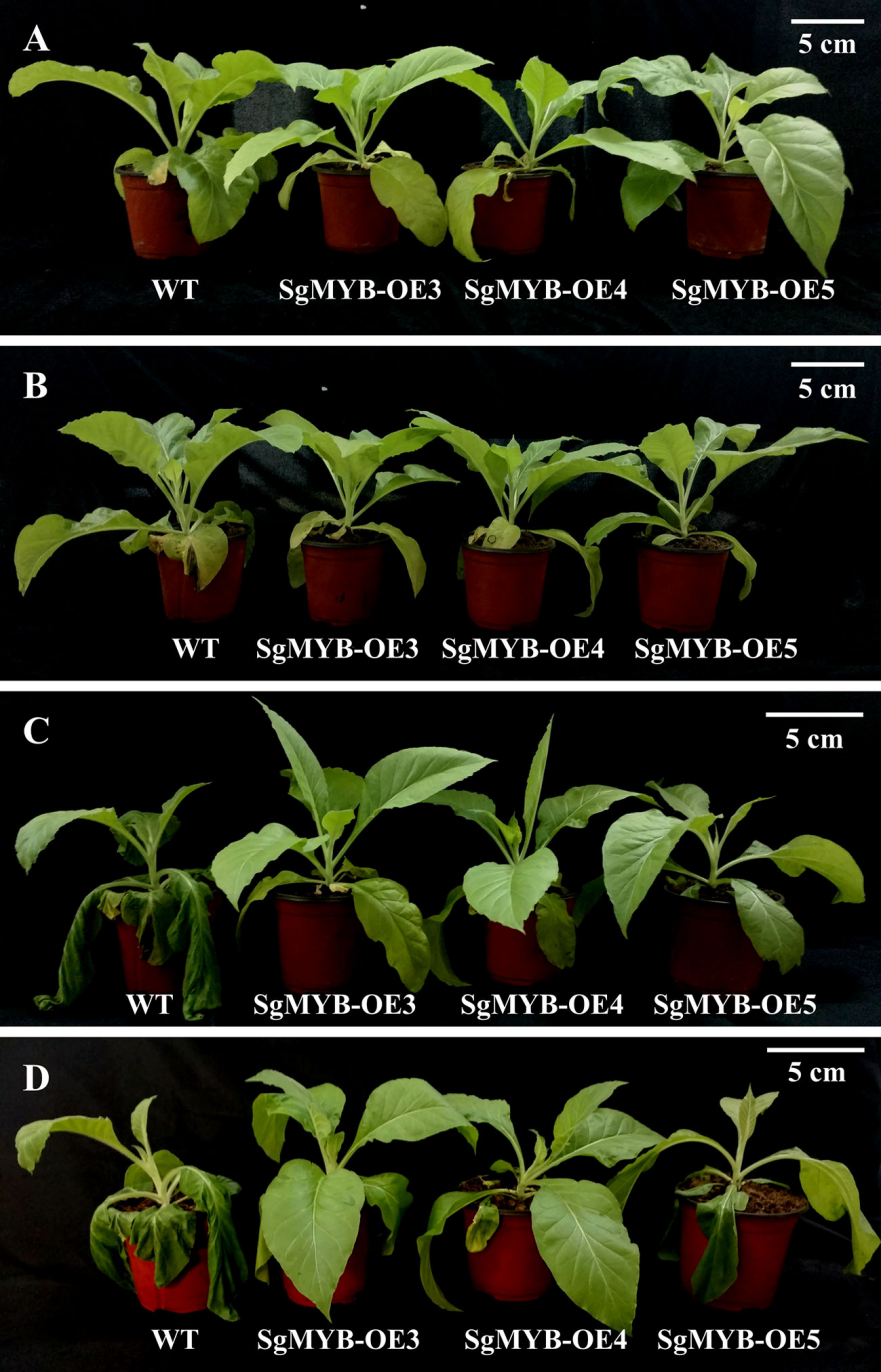
Improved cold tolerance of *SgMYB* overexpressing transgenic tobacco lines (SgMYB-OE3, SgMYB-OE4 and SgMYB-OE5) compared with wide-type tobacco (WT). All the tobacco plantlets were exposed to cold treatment at 4°C and 70% humidity under a 16/8-h (light/dark) photoperiod. The photos were taken before treatment **(A)**, and after cold treatment of 12 **(B)**, 24 **(C)** and 48 **(D)** hours. The white bar stands for 5cm.

Transgenic (*SgRVE6*-OE3, *SgRVE6*-OE4, and *SgNAC2*-OE5) and wild-type (WT) tobacco plants in eight-leaf stage were exposed to cold treatment at 4°C and 70% humidity under a 16/8-h (light/dark) photoperiod. Different levels of wilting symptoms were observed between transgenic and wild-type leaves after 24 h of cold treatment. All the three *SgRVE6*-OE lines showed better tolerance to cold stress than the wild-type (**Figure 4**).

**Figure 5:**
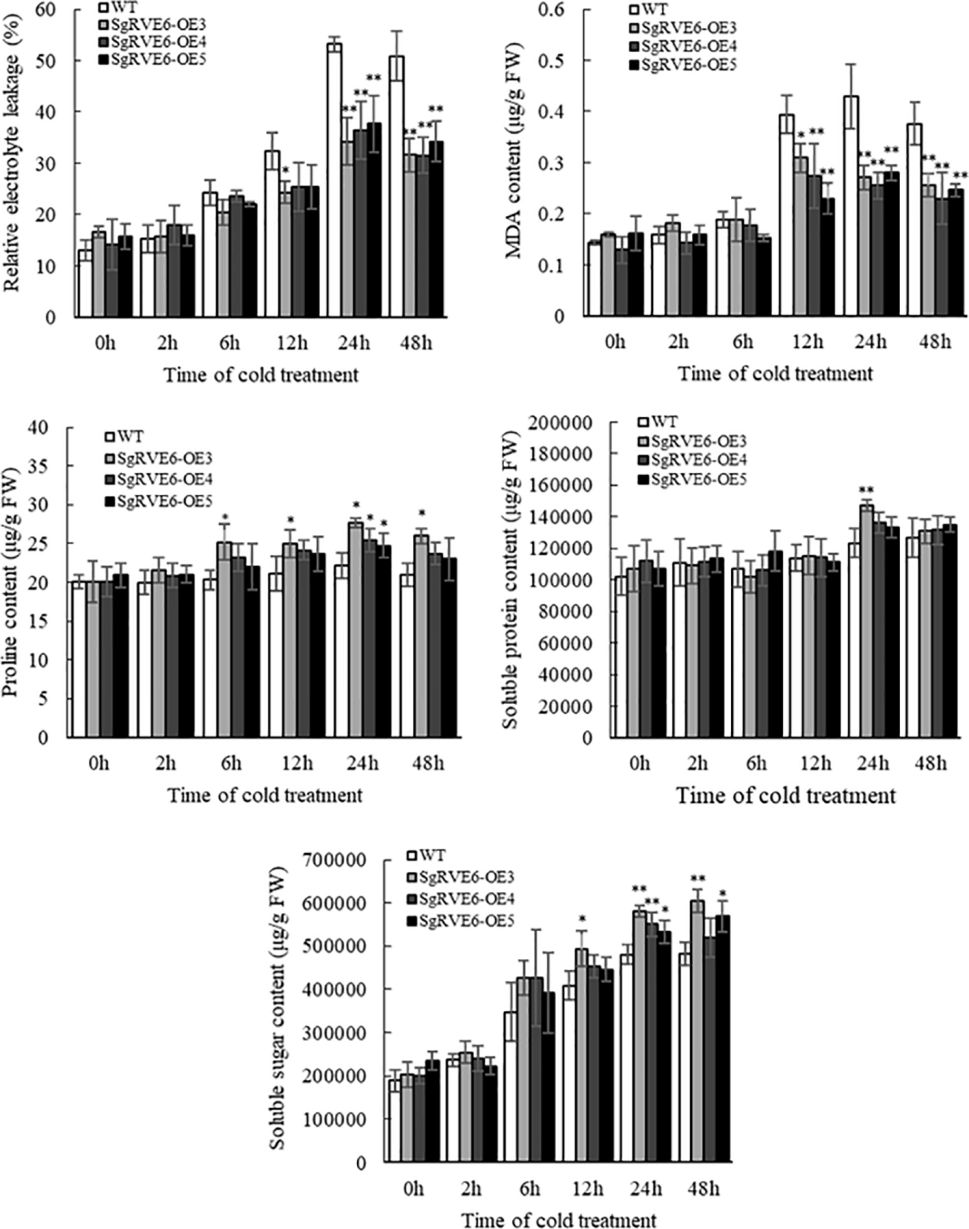
Physiological variations of SgRVE6 over-expressing transgenic (SgRVE6-OE3, SgRVE6-OE4 and SgRVE6-OE5) and wild-type (WT) tobacco under normal and cold conditions. **(A)** Relative electrolyte leakage. **(B)** MDA content. **(C)** Proline content. **(D)** Soluble protein content. **(E)** Soluble sugar content. The physiological measurements were represented as means ± SD of three sample replicates. * and ** above the columns indicated significant difference at *P*<0.05 and *P*<0.01 with WT.

To further determine the enhanced cold tolerance in transgenic lines, stress-related physiological parameters were compared between transgenic and WT plants under normal and cold conditions. Under normal growth condition, there was no significant difference in any physiological parameter between transgenic and WT plants. When the plants were subjected to cold treatment, all the physiological parameters were enhanced in both transgenic and WT plants, but no significant difference was observed between transgenic and WT plants at first. As the cold treatment continued, the three *SgRVE6*-OE lines started to show enhanced physiological responses compared to the WT line, including significantly lower MDA content and relative electrolyte leakage, and significantly higher proline, soluble sugar and soluble protein contents, especially the *SgRVE6*-OE3 line which was significantly better than the WT line in all the five physiological parameters (**Figure 5**).

### DEGs in SgRVE6 Over-Expressing Tobacco

**Figure 6:**
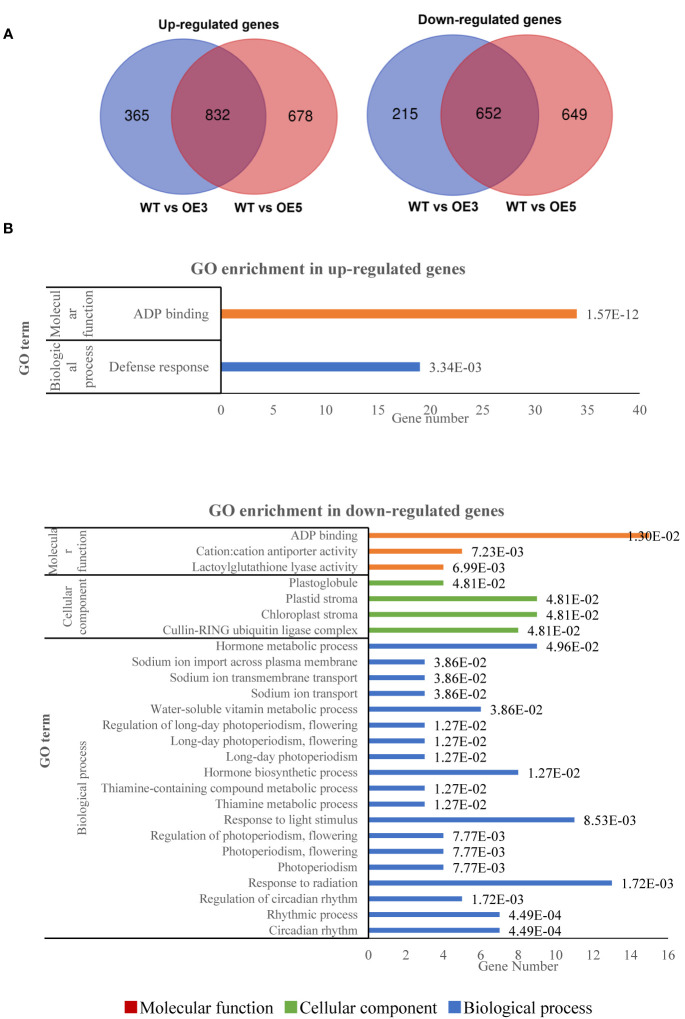
Venn diagram **(A)** and GO enrichment **(B)** of up-regulated and down-regulated genes shared by *SgRVE6*-OE3 and *SgRVE6*-OE5 tobacco lines.

The RNA-seq analysis showed that no read was mapped *SgRVE6* in WT, and an average of 4432 and 4612 reads were mapped to *SgRVE6* in *SgRVE6*-OE3 and 5, respectively, proving that *SgRVE6* were over-expressed in *SgRVE6*-OE3 and 5. Compared with WT, 2064 (1197 up-regulated and 867 down-regulated) and 2811 (1510 up-regulated and 1301 down-regulated) genes were identified as DEGs in *SgRVE6*-OE3 and *SgRVE6*-OE5, respectively. 832 up-regulated and 652 down-regulated genes were shared between *SgRVE6*-OE3 and *SgRVE6*-OE5, and therefore identified as genes induced and repressed by SgRVE6, respectively (**Figure 6A**). GO enrichment analysis indicated that the SgRVE6-induced genes were enriched in GO terms of “ADP binding” and “Defense response”, and the SgRVE6-repressed genes in 26 GO terms, among which 10 GO terms were associated with circadian rhythm, photoperiodism and response to light stimulus (**Figure 6B**). KEGG enrichment analysis indicated that the SgRVE6-repressed genes were enriched in “circadian rhythm” pathway (ID: nta04712). KEGG module enrichment analysis indicated that the SgRVE6-induced genes were enriched in the model “tryptophan biosynthesis, chorismate => tryptophan” (ID: M00023).

### SgRVE6 Modulates the Expression of Circadian Clock Genes

**Figure 7:**
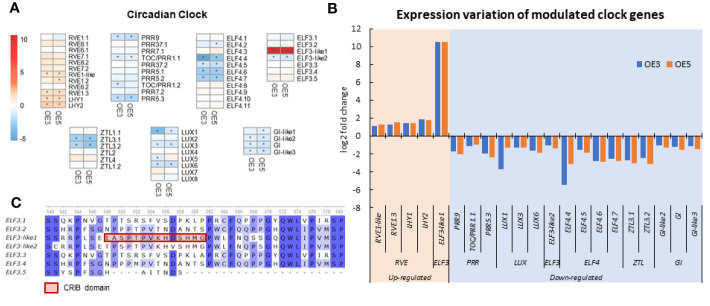
Expression variation of circadian clock genes in *SgRVE6*-OE3 and *SgRVE6*-OE5 tobacco lines. **(A)** Heatmap representing circadian clock gene expression variation (log_2_ fold change) of in *SgRVE6*-OE3 and 5; **(B)** Expression variation of expression variation (log_2_ fold change) of circadian clock genes up-regulated and down-regulated in *SgRVE6*-OE3 and 5; **(C)** Partial alignment of tobacco ELF protein sequences showing a unique CRIB domain in ELF-like1. The heatmap cells with |log2FC| > 1 were marked with *.

According to the GO and KEGG enrichment analysis, the SgRVE6-supressed genes were enriched in several GO terms and a pathway associated with circadian rhythm. Thus, to further investigate the impact of SgRVE6 on circadian rhythm of tobacco, the expression profiles of 58 circadian clock genes were extracted from the whole transcriptome survey through BLAST search with *A. thaliana* clock genes (Linde et al., 2017), including 12 *RVEs*, 10 *PSEUDO-RESPONSE REGULATORs* (*PRRs*), 8 *LUX ARRHYTHMOs* (*LUXs*), 7 *EARLY FLOWERING3s* (*ELF3s*), 11 *EARLY FLOWERING4s* (*ELF4s*), 6 *ZEITLUPE* (*ZTLs*), and 4 *GIGANTEAs* (*GIs*) (**Figure 7A**). A total of 16 genes were down-regulated (FDR < 0.05 and log_2_FC < −1) in both OE3 and OE5 lines, including 3 *PRRs*, 4 *ELF4s*, 1 *ELF3s*, 2 *ZTLs*, 3 *LUXs*, and 3 *GIs*. Five genes were up-regulated (FDR < 0.05 and log_2_FC > 1) in both OE3 and OE5 lines, including 4 *RVEs* and 1 *ELF3* (**Figure 7B**). The only up-regulated ELF3-clade gene, *ELF3-like1*, contains a unique CRIB domain and showed a very high log_2_FC value of 10.5 (**Figures 7B, C**).

### SgRVE6-Inducible Genes in Response to Cold Stress

**Figure 8:**
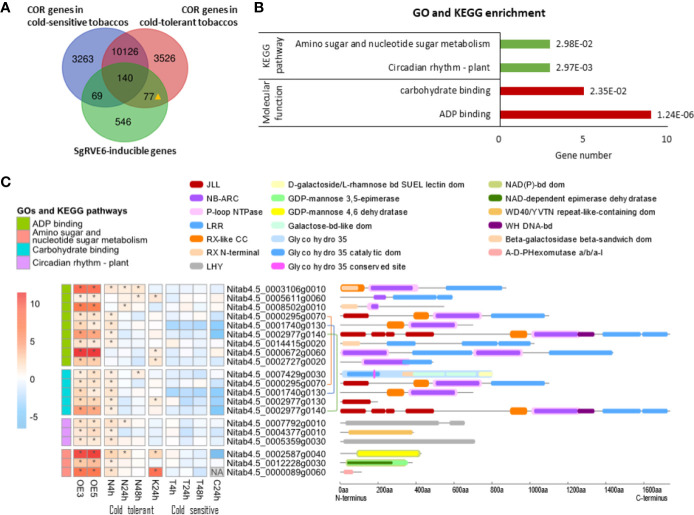
Expression profiling, GO and KEGG pathway enrichment analysis of cold-tolerant exclusive, SgRVE6-inducible cold-responsive (COR) genes in tobaccos. **(A)** Venn diagram of SgRVE6-inducible genes and COR genes in cold-sensitive (NC567 and K326) and cold-tolerant (Taiyan8 and CB-1) tobaccos; 77▲ indicates the 77 genes which were SgRVE6 inducible and also exclusively expressed in cold-tolerant tobaccos; **(B)** GO and KEGG enrichment analysis of the 77 cold-tolerant exclusive, SgRVE6-inducible genes. **(C)** Heatmap of expression variations and InterPro annotation of the cold-tolerant exclusive, SgRVE6-inducible genes associated with enriched GOs and KEGG pathways; OE3 and OE5 indicate the log_2_ fold change (log_2_FC) values of gene expressions in *SgRVE6*-OE3 and *SgRVE6*-OE5 tobacco lines compared to W38, respectively; N, K, T and C represent NC567, K326, Taiyan8 and CB-1 tobaccos, respectively; 4, 24, and 48 h indicate the log_2_FC values of gene expressions in tobaccos after 4-, 24-, and 48-h cold treatments; the heatmap cells with log2FC > 1 were marked with *; The genes connected with the same-color line were one gene associated with both “ADP binding” and ‘carbohydrate binding’.

To explore the role of the SgRVE6-inducible genes in cold responses, sequencing data of two previous whole-transcriptome surveys on tobaccos under cold stress were used in our research: (1) gene expression profiling of cold-tolerant NC567 and cold-sensitive Taiyan8 after 4, 24, and 48 h cold treatment (N4h, N24h, N48h, T4h, T24h and T48h) (Hu et al., 2016); (2) gene expression profiling of cold-tolerant K326 and cold-sensitive CB-1 after 24-h cold treatment (K24h and C24h) (Wang et al., 2014). The up-regulated genes in N4h, N24h N48h, and K24h were referred as *COLD REGULATED* (*COR*) genes in cold-tolerant tobaccos, and those in T4h, T24h, T48h, and C24h were referred as *COR* genes in cold-sensitive tobaccos. Comparing them with SgRVE6-inducible genes, 26% (217) of the SgRVE6 inducible genes were found to be cold responsive in cold-tolerant tobaccos, 25% (209) in cold-sensitive tobaccos, and 77 SgRVE6-inducible genes were found to respond to cold stress only in cold-tolerant tobaccos and referred to as cold-tolerant exclusive, SgRVE6-inducible (CTE-SI) genes (**Figure 8A**). GO and KEGG enrichment analysis indicated that these 77 CTE-SI genes were enriched in two GO categories (“ADP binding” and “Carbohydrate binding”) and two KEGG pathways (“Amino sugar and nucleotide sugar metabolism” and “Circadian rhythm-plant”). A total of 17 CTE-SI genes were found from the two GO categories and KEGG pathways, 9 were associated with “ADP binding,” 5 with “carbohydrate binding,” 3 with both GO categories, 3 with “Amino sugar and nucleotide sugar metabolism” and 3 with “Circadian rhythm-plant” (**Figures 8B, C**). Among the nine “ADP binding” CTE-SI proteins, 8 contained NB-ARC domain, 7 contained LRR domain and 6 contained both NB-ARC and LRR domains. Among the 5 “Carbohydrate binding” CTE-SI proteins, 3 contained JLL domain and 3 contained NB-ARC domain. Two genes of “Circadian rhythm-plant” contained LHY domain (**Figure 8C**), and were referred to as LHY1 and LHY2 in **Figure 7A**.

## Discussion

Low temperature is the major abiotic constraint on the cultivation and production of stylo (Chakraborty, 2004). Most stylo species were not able to survive through winter in the northern regions of Guangdong province (25°N 114°E), except for fine-stem stylo (*S. guianensis* var. *intermedia*), which remains green the entire winter. The superior cold tolerance makes fine-stem stylo a valuable material to study the genetic networks and molecular mechanisms underlying stylo’s physiological responses and adaptive systems to counteract cold stress. Transcriptomic profiling of fine-stem stylo under normal and cold conditions was conducted by our lab, which revealed that a *RVE* gene was significantly over-expressed under cold stress and therefore selected as the candidate gene to study cold responsive networks and adaptation mechanisms in fine-stem stylo.

The RVE transcription factor family is recently found to be a new amendment to the classic circadian clock loop in *A. thaliana* (Farinas and Mas, 2011; Rawat et al., 2011; Hsu et al., 2013; Fogelmark and Troein, 2014). The 11 members of the RVE family in *A. thaliana* (CCA1, LHY and nine RVEs) all encode proteins with a single highly conserved MYB/SANT domain containing a SHAQKYF motif (Farinas and Mas, 2011; Rawat et al., 2011). The *A. thaliana* RVE family consists of two subgroups, one comprising CCA1, LHY, RVE1, 2, 7 and RVE7-like and the other RVE3, 4, 5, 6 and 8. The second subgroup, referred to as the LCL subfamily, shares an additional conserved region (LCL domain) outside the MYB domain (Farinas and Mas, 2011). The alignment indicated that SgRVE6 shares similar sequence features (a SHAQKYF-class MYB domain and a LCL domain) with the LCL subfamily in *A. thaliana*. Phylogenetic analysis also clustered SgRVE6 to the LCL subgroup in RVE family.

RVE4, RVE6 and RVE8 have been reported as positive regulators of clock genes including *TIMING OF CAB EXPRESSION1* (*TOC1*), the *PRRs* and the evening complex (EC) genes (*ELF3*, *ELF4*, and *LUX*) in *A. thaliana* (Farinas and Mas, 2011; Rawat et al., 2011; Hsu et al., 2013). However, in our research, more circadian clock genes were down-regulated than those were up-regulated by SgRVE6 in tobacco OE lines (**Figure 7**). A possible explanation for this result, which contradicts previous research, is that the clock genes were not down-regulated through direct interaction with SgRVE6, but indirectly through some intermediate regulators induced by SgRVE6, such as LHY/CCA1. The morning expressed MYB-like transcription factors LHY/CCA1 are repressors of the afternoon expressed *PRR* genes (*PRR1/TOC1*, *PRR5*, *PRR7*, and *PRR9*) (Alabadí et al., 2001; Farré et al., 2005; Kamioka et al., 2016), EC genes (Huang and Nusinow, 2016), and *GIs* (Lu et al., 2012). In our research, two tobacco *LHY* genes were significantly up-regulated and the elevated expression of the *LHY*s might be responsible for inhibiting the expression of *PRRs*, *EC* components and *GIs* in SgRVE6-OE lines. Among the up-regulated clock genes, the expression of *ELF3-like1* showed a very high log_2_FC value of 10.5 (**Figure 8B**), while the expression of other ELF3-clade genes had either no significant variation or a moderate decrease (**Figure 8A**). The difference between ELF-like1 and the other ELF3-clade proteins is that ELF-like1 contained a Cdc42/Rac interactive binding (CRIB) domain (**Figure 8C**). CRIB domain is the most conserved region of a GTPase binding domain (GBD)/p21 binding domain (PBD), which usually exists in many downstream effectors of the small GTPases Cdc42 and Rac (Swaminathan et al., 2014). This unique CRIB domain might be responsible for the different expression variation of *ELF-like1* from the other ELF3 clade genes, and therefore could be a breakthrough point for the further research on interaction between SgRVE6 and ELF3.

In plants, the role of circadian clock extends to almost every aspect of growth and development, including responses to abiotic stresses (Nagel et al., 2015). The circadian clock in *A. thaliana* exerts a critical role in timing multiple biological processes, including stress responses, through the regulation of up to 80% of the transcriptome. A few studies have reported that *CCA1/LHY/RVE* mutants could change sensitivity to abiotic stresses in *A. thaliana*: a *cca1/lhy* mutant exhibited greater sensitivity to salt, osmotic and heat stress (Cao et al., 2005; Kant et al., 2008), while a *rve1* mutant showed greater tolerance to low temperature (Meissner et al., 2013). As a member of the RVE family, SgRVE6 might also participate in the regulation of abiotic stress responses in plant species. A previous RNA-seq analysis conducted by our lab revealed that the expression of *SgRVE6* in fine-stem stylo was significantly upregulated when subjected to cold stress. In this paper, we further confirmed the expression patterns of *SgRVE6* under cold treatment with Real-time PCR. The results showed that *SgRVE6* was universally expressed in root, stem and leaf tissues of fine-stem stylo, but most abundant in leaf. Although the *SgRVE6* expression level was different among root, stem and leaf, their expression variations under cold stress were similar. The *SgRVE6* expressions in different tissues all peaked after 2 h of cold treatment and stayed at the high expression level as cold treatment continued (**Figure 3**), implying a persistent role of SgRVE6 in the regulation of cold responses in fine-stem stylo. Thus, an over-expressing vector carrying *SgRVE6* CDS was transferred into tobacco through *Agrobacterium*-mediated transformation to further investigate the molecular function of SgRVE6 in plants. All the transgenic tobaccos over-expressing *SgRVE6* showed higher tolerance to cold treatment than the wild-type tobaccos (**Figure 4**). The differences of physiological changes between transgenic and wild-type tobaccos also supported this result (**Figure 5**). As the cold treatment extended, lower levels of relative electrolyte leakage and MDA were accumulated in transgenic tobaccos, indicating less membrane damage and cold injury (Xiong et al., 2002; Chen et al., 2012); higher levels of proline, soluble protein and soluble sugar were accumulated in transgenic tobaccos (Jouve et al., 1993; Peng et al., 2016; Yu et al., 2017). All these results demonstrated that SgRVE6 could promote the physiological system in plants to counteract cold stress and therefore improve their cold tolerance.

Increasing evidence has proven that the mechanisms of cold acclimation involve the convergence of circadian regulation and cold responses. The C-REPEAT BINDING FACTOR/DEHYDRATION-RESPONSIVE ELEMENT-BINDING (CBF/DREB) dependent, cold-response pathway is a canonical mechanism responsible for cold acclimation. CBF proteins activate the transcription of a substantial number of *COR* genes, and enhance cold tolerance through re-establishment of hormone homeostasis, synthesis of cryoprotective protein, accumulation of primary and secondary metabolites. It has been clarified that *CBF* gene expression is regulated by several major clock components. CCA1 and LHY directly activate the transcription of *CBF1*, *CBF2* and *CBF3* genes by binding to their promoters (Dong et al., 2011), while PRR9/7/5 proteins negatively regulate the expression of *CBF* genes and their downstream *COR* genes (Nakamichi et al., 2009; Nakamichi et al., 2012). Phytochrome-Interacting Factor7 (PIF7) protein, along with the clock component TOC1/PRR1, also represses the expression during the subjective night by binding to a G-box of the CBF2/DREB1C promoters (Kidokoro et al., 2009). A clock component can also regulate cold responses in a CBF-independent manner. Higher susceptibility to freezing stress was observed in the *GI*-deficient mutant *gi-3* without alteration of *CBF* expression levels, indicating that GI confers freezing tolerance without the mediation of CBF proteins (Cao et al., 2005). In our study, over-expression of *SgRVE6* in tobacco altered the expression of several clock components that were proven to regulate *CBF* transcription in *Arabidopsis*, e.g., activators LHY1 and LHY2 were upregulated, and repressors PRR1.1, PRR5.3 and PRR9 were downregulated (**Figures 7A, B**), yet no significant variation was observed in the expression of *CBF* genes of *SgRVE6*-OE tobaccos (**Supplementary Table 5**). It is likely that SgRVE6, like GI, regulates cold tolerance in a CBF independent manner, but the reason that the transcriptional variations in LHYs and PRRs did not alter the expression of *CBF* genes in tobacco remains unclear and needs further investigation. A possible explanation is that the transcriptional activation of *CBF* genes by LHYs and PRRs in tobacco is more complicated, and requires other co-activators or environmental signals.

Emerging evidence indicates that there exists a signaling crosstalk between cold response and pathogen defense in plants. It has been reported that cold exposure prior to pathogen inoculation efficiently enhanced pathogen resistance in *A. thaliana* (Seo et al., 2010; Singh et al., 2014; Kim et al., 2017; Wu et al., 2019). Furthermore, several *A. thaliana* mutants with enhanced pathogen immunity exhibited altered cold tolerance as well, including *chs3*, *cpr1-1*, *cpr5-2*, and *slh1* mutants with increased cold tolerance (Yang et al., 2010), and *chs2*/*rpp4*, *bon1-1*, and *snc1-1* with increased cold sensitivity (Huang et al., 2010). Among these mutants, *chs3* and *slh1* shared a similar intra-molecular modulation. CHS3 and SLH1 proteins both have a nucleotide NB-LRR domain at the N-terminus and a special domain in the C-terminus (a LIM domain in CHS3 and a WRKY domain in SLH1). The special domain modulates the protein activity through intra-molecular interaction with its own NB-LRR domain. In *chs3* and *slh1*, the mutations in the special C-terminus domain blocked the intra-molecular interaction and led to constitutive activation of the N-terminus NB-LRR domain, which conferred enhanced pathogen immunity as well as freezing tolerance to the mutants. These findings revealed a role of NB-LRR proteins in the mutual interaction between cold signaling and defense responses. In our research, 77 genes were found to be induced by SgRVE6 and also only respond to cold stress in cold-tolerant tobaccos, and 6 of these genes contained NB-LRR domain, which took up to 8% of the CTE-SI genes and led to the enrichment in “ADP binding” and “Carbohydrate binding”. These results suggest that the NB-LRR genes are most likely the main reason for the elevated cold tolerance in SgRVE6-OE lines.

In conclusion, SgRVE6 is a transcription factor that belongs to the LCL subgroup of RVE family. *SgRVE6* is universally expressed in root, stem and leaf tissues of fine-stem stylo, and highly responsive to cold stress. Over-expressing *SgRVE6* can significantly improve the cold tolerance of tobacco plants. RNA-seq analysis revealed that over-expression of *SgRVE6* significantly affected expression of circadian clock genes and induced cold-tolerant exclusive NB-LRR genes in tobacco. The results imply that SgRVE6 functions in a CBF independent manner, and the cold-tolerant exclusive NB-LRR genes play a key role in SgRVE6 regulation of cold tolerance in tobacco. Future work will need to verify the interaction of SgRVE6 and NB-LRR genes, and explore the function of NB-LRR proteins in cold responses.

## Data Availability Statement

The datasets generated for this study can be found in the NCBI, SRA database, PRJNA316912, PRJNA608154, PRJNA368913, PRJNA287250, PRJNA277095.

## Author Contributions

X-MX conceived and initiated the project. SC, H-AH, and J-HC designed and performed most of the experiments and data analysis. SC and H-AH wrote the article. C-CF, P-LZ, S-WK, and X-QZ participated in plasmid construction, plant transformation and physiological determination. SC, J-HC, and T-XZ conducted the bioinformatic analysis. All authors contributed to the article and approved the submitted version.

## Conflict of Interest

The authors declare that the research was conducted in the absence of any commercial or financial relationships that could be construed as a potential conflict of interest.

## Abbreviations

RVE, REVEILLE; NB, nucleotide binding domain; LRR, leucine-rich repeats; CCA1, CIRCADIAN CLOCK ASSOCIATED 1; LHY, LATE ELONGATED HYPOCOTYL; LCL, LHY/CCA1-LIKE; TOC1, TIMING OF CAB EXPRESSION1; PR, PSEUDO-RESPONSE REGULATOR; LUX, LUX ARRHYTHMO, ELF EARLY FLOWERING; ZTL: ZEITLUPE; GI: GIGANTEA; MS medium, Murashige and Skoog medium; BA, Benzylaminopurine; NAA, 1-Naphthylacetic acid; WT, Wild-type; cDNA, complementary DNA; DEG, differentially expressed gene.
