# Supplementary material for: SgRVE6, a LHY-CCA1-Like Transcription Factor From Fine-Stem Stylo, Upregulates NB-LRR Gene Expression and Enhances Cold Tolerance in Tobacco
Source: Front Plant Sci. 2020 Aug 19;11:1276. doi: 10.3389/fpls.2020.01276 (PMC7466579; doi:10.3389/fpls.2020.01276)
Supplement: Supplementary file 1 [file DataSheet_1.docx]

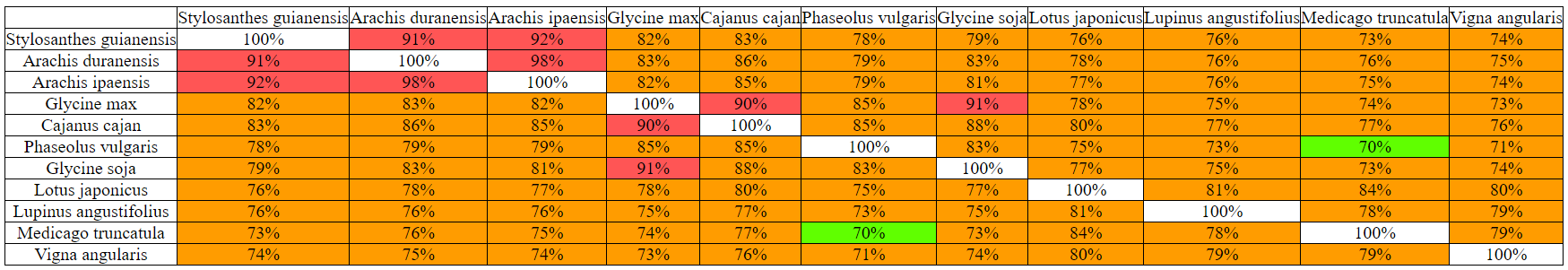


**Supplementary Figure 1** Percent identities between the RVE protein sequences of the leguminous species.


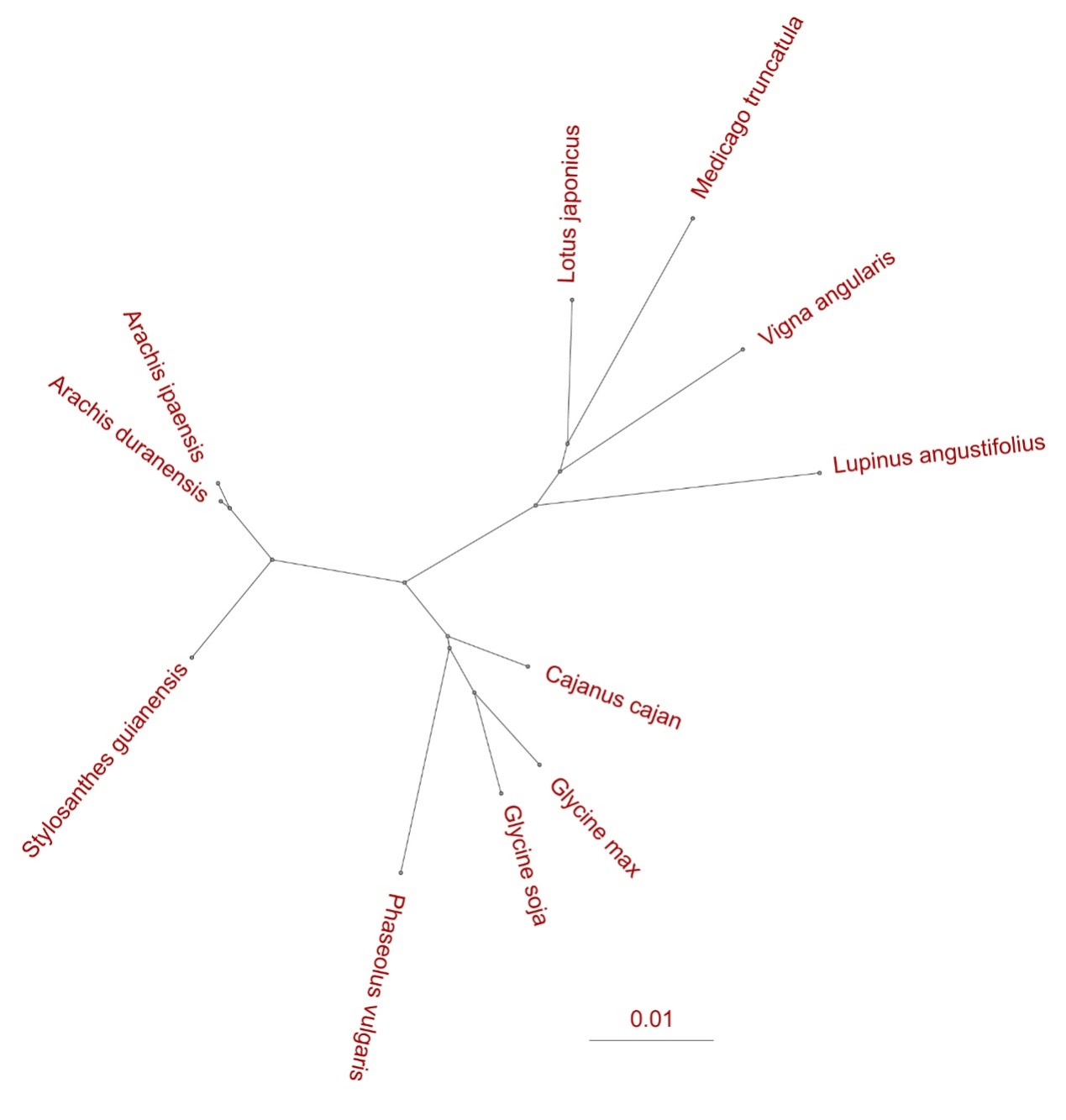


**Supplementary Figure 2** Phylogenetic analysis of the RVE6 protein sequences of the leguminous species. The oval delimited the cluster containing the *Stylosanthes guianensis* RVE6, SgRVE6.


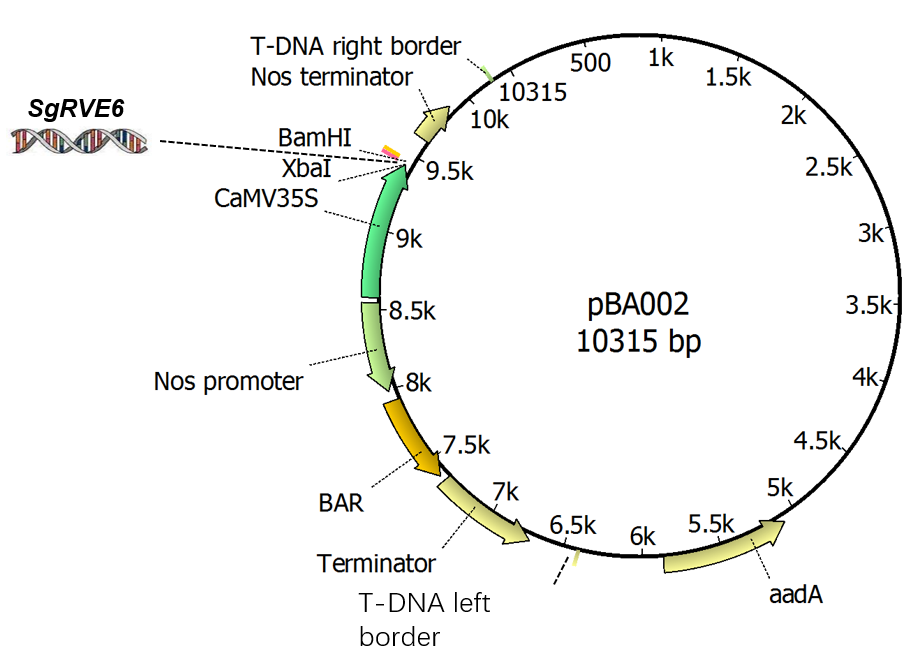


**Supplementary Figure 3** Schematic representation of pBA002 over-expression vector used for genetic transformation. The *SgRVE6* CDS was inserted between XbaI and BamHI restriction enzyme sites; BAR represents *bar* gene and confers Basta herbicide resistance to plants; *aadA* confers spectinomycin resistance.


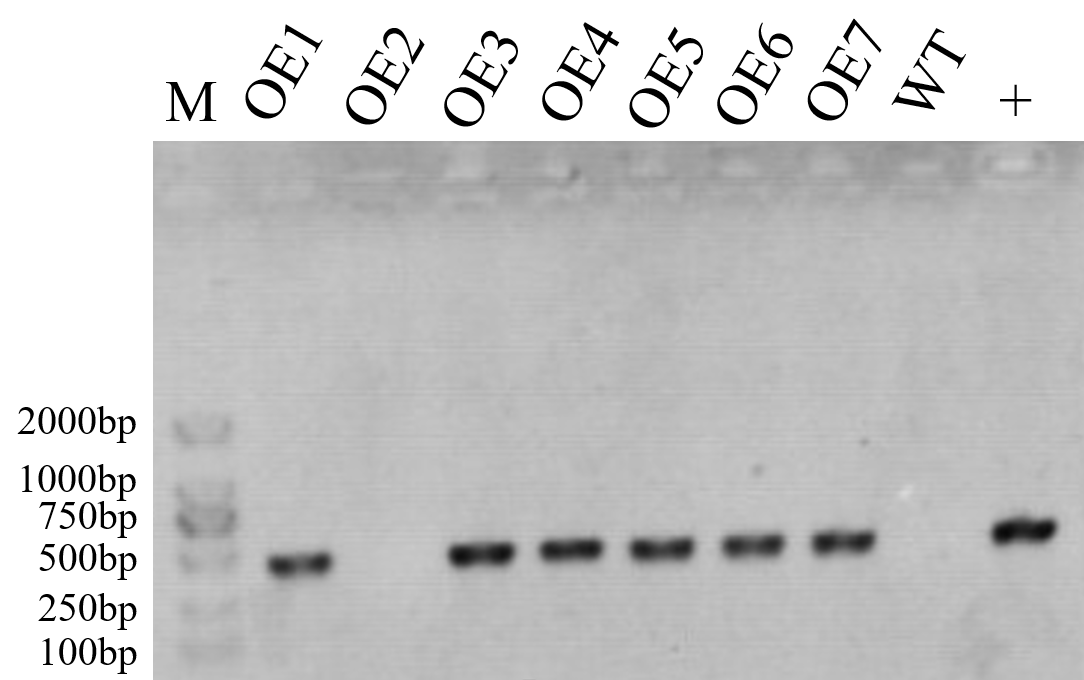

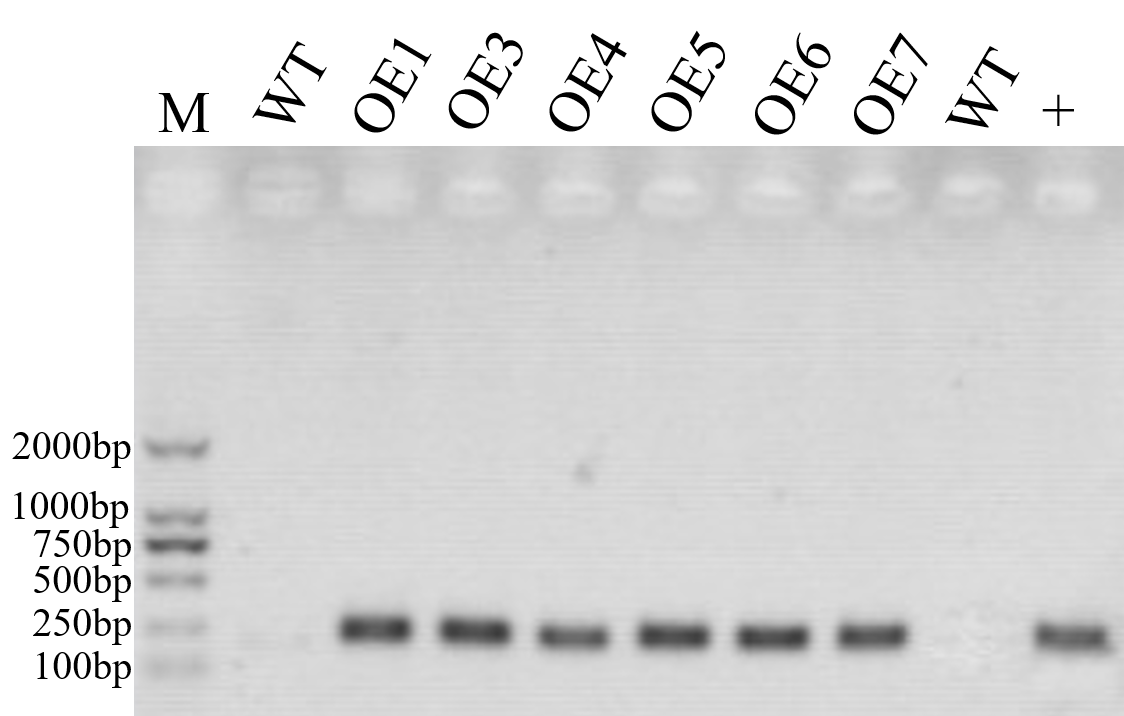


**D**

**C**

**A**

**B**


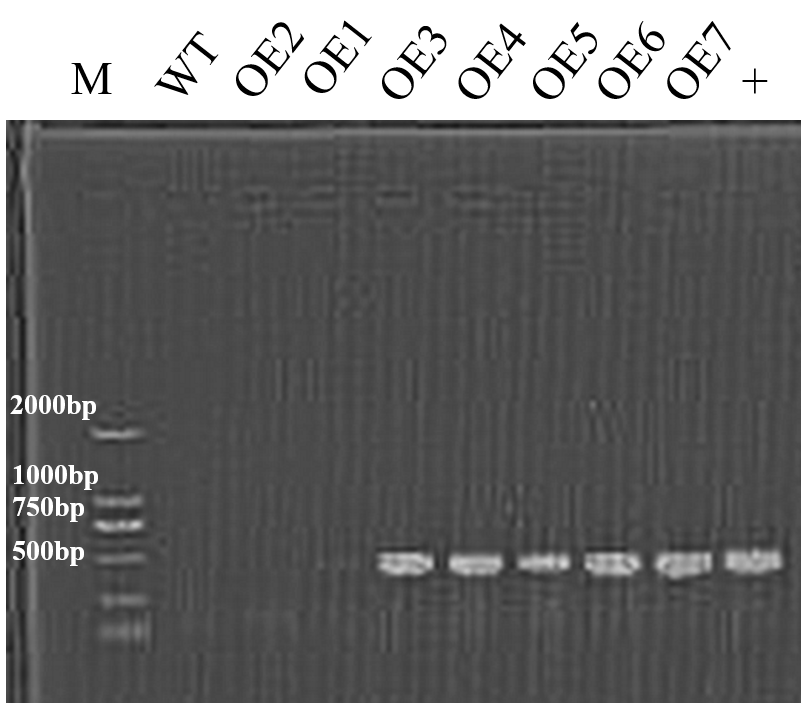

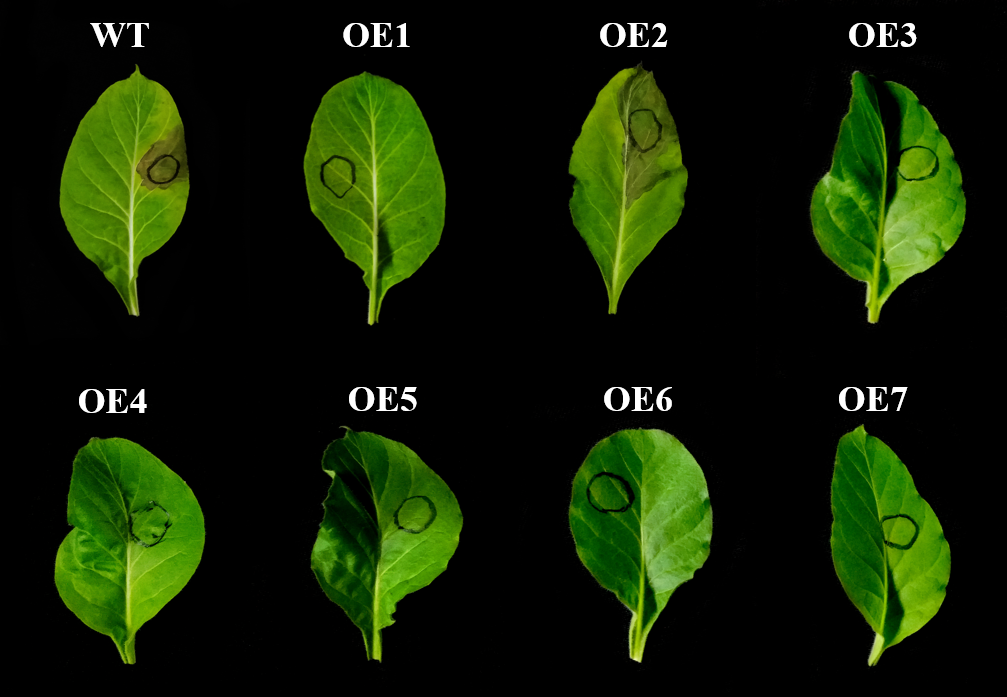


**Supplementary Figure 4** Verification of *SgRVE6* over-expressing transgenic tobaccos lines by amplification of *bar* gene (A) from genomic DNA, amplification of *SgRVE6* from genomic DNA (B) and cDNA (C), and foliar application of basta (D).

(A and B) Genomic DNA was extracted from wild-type (WT) and *SgRVE6* over-expressing transgenic lines (OE1-7); The extracted DNAs were amplified with two primer pairs, *bar*-F and *bar*-R, *SgRVE6*-F1 and *SgRVE6*-R1 (Supplementary Table 1); (C) Total RNA was extracted from WT and *SgRVE6-*OE1-7, and used to synthesize cDNA for PCR amplification with primer pair, *SgRVE6*-F2 and *SgRVE6*-R2 (Supplementary Table 1), M indicated molecular markers, + represented the plasmid used in corresponding gene transformation and served as a positive control, WT served as a negative control; (D) 0.2% (w/v) basta was painted on black circle area of WT and *SgRVE6*-OE leaves.

**Supplementary Table 1** The primer pairs used in gene cloning, plasmid construction and Real-time PCR

| Primer | Primer sequences |
| --- | --- |
| *SgRVE6*-F | ATGAACCCGAACCCGTCAGAGGGATTG |
| *SgRVE6*-R | TACTTGGGCTTGGGCAGTCTCCCTAAC |
| *SgRVE6*-PBA-F | ACGCGTATGAACCCGAACCCGTCAGAG |
| *SgRVE6*-PBA-R | ACTAGTCTAAATAGCTGTCTTCAGCC |
| *bar*-F | ATGCCTCTGCCGACAGTGGT |
| *bar*-R | GGGAAATTCGAGCTCACTAG |
| *SgRVE6*-F1 | AGAAGAGCGGGACAAGTGAA |
| *SgRVE6*-R1 | TGACCAGGAAGACATTGCAG |
| *SgRVE6*-F2 | GTCCTGCAGCCTGATTCTTC |
| *SgRVE6*-R2 | CACCAGGCATGGGTCTATCT |

**Supplementary Table 2** The names, species and accession numbers of the 14 leguminous RVE proteins

| Name | Species | Accession number |
| --- | --- | --- |
| AdRVE6 | *Arachis duranensis* | XP_015941336.1 |
| AiRVE6 | *Arachis ipaensis* | XP_016176733.1 |
| GmRVE6 | *Glycine max* | XP_006589522.1 |
| CcRVE6 | *Cajanus cajan* | XP_020236024.1 |
| PvRVE6 | *Phaseolus vulgaris* | XP_007143469.1 |
| GsRVE6 | *Glycine soja* | KHN12936.1 |
| LjRVE6 | *Lotus japonicus* | AFK39021.1 |
| LaRVE6 | *Lupinus angustifolius* | XP_019454354.1 |
| MtRVE6 | *Medicago truncatula* | XP_013442632.1 |
| ZjRVE6 | *Ziziphus jujuba* | XP_015878398.1 |
| VaRVE6 | *Vigna angularis* | XP_017437480.1 |
| JrRVE6 | *Juglans regia* | XP_018847295.1 |
| TcRVE6 | *Theobroma cacao* | EOY23319.1 |
| NnRVE6 | *Nelumbo nucifera* | XP_010261345.1 |

**Supplementary Table 3** The names and *Arabidopsis* Genome Initiative (AGI) numbers of the 11 members of the RVE family in *Arabidopsis thaliana*

| **Name** | **AGI number** |
| --- | --- |
| CCA1 | At2g46830 |
| LHY | At1g01060 |
| RVE1 | At5g17300 |
| RVE2/CIR1 | At5g37260 |
| RVE3 | At1g01520 |
| RVE4 | At5g02840 |
| RVE5 | At4g01280 |
| RVE6 | At5g52660 |
| RVE7/EPR1 | At1g18330 |
| RVE7-like | At3g10113 |
| RVE8 | At3g09600 |

**Supplementary Table 4** Count of reads mapped to *SgRVE6* in wild-type, *SgRVE6*-OE3 and *SgRVE6*-OE5 tobacco lines

| Tobacco line | Replicate 1 | Replicate 2 | Replicate 3 | Means | log_2_FC^1^ |
| --- | --- | --- | --- | --- | --- |
| Wild-type | 0 | 0 | 0 | 0 |  |
| *SgRVE6*-OE3 | 2199.353 | 5309.537 | 5788.27 | 4432.387 | 14.5443 |
| *SgRVE6*-OE5 | 4193.34 | 4795.072 | 4850.135 | 4612.849 | 14.54558 |

1 log_2_FC means the log_2_ fold changes of RPKM values detected from the RNA-seq results of SgRVE6-OE3 and SgRVE6-OE5 compared with wild-type tobacco.

**Supplementary Table 5** Expression variations of *CBF* genes in SgRVE6 over-expressing transgenic tobaccos

| Tobacco Gene ID^1^ | Top Hit Accession^2^ | Top Hit Annotation^2^ | OE3_log_2_FC^3^ | OE3_padj^4^ | OE5_log_2_FC^3^ | OE5_padj^4^ |
| --- | --- | --- | --- | --- | --- | --- |
| Nitab4.5_0001083g0010 | AT4G25490.1 | C-repeat/DRE binding factor 1 | -1.095480949 | 0.57152142 | 0.571976802 | 0.721941367 |
| Nitab4.5_0002394g0010 | AT4G25470.1 | C-repeat/DRE binding factor 2 | -0.874683039 | 0.630894114 | 0.729969149 | 0.627149281 |
| Nitab4.5_0002419g0020 | AT4G25470.1 | C-repeat/DRE binding factor 2 | 3.487344312 | 0.37710442 | 2.384958549 | 0.549931612 |
| Nitab4.5_0002419g0050 | AT5G51990.1 | C-repeat-binding factor 4 | -2.001328251 | 0.33386219 | 0.16654031 | 0.885431211 |
| Nitab4.5_0002419g0060 | AT5G51990.1 | C-repeat-binding factor 4 | NA | NA | 1.61766985 | 0.689286081 |
| Nitab4.5_0002914g0060 | AT4G25490.1 | C-repeat/DRE binding factor 1 | NA | NA | NA | NA |
| Nitab4.5_0003529g0010 | AT5G51990.1 | C-repeat-binding factor 4 | -2.517053333 | 0.435391882 | 0.520671324 | 0.815260451 |
| Nitab4.5_0003529g0020 | AT4G25490.1 | C-repeat/DRE binding factor 1 | NA | NA | NA | NA |
| Nitab4.5_0005990g0020 | AT4G25490.1 | C-repeat/DRE binding factor 1 | -1.017150654 | 0.656206111 | 0.918976996 | 0.640001023 |
| Nitab4.5_0005990g0030 | AT5G51990.1 | C-repeat-binding factor 4 | -3.241083744 | 0.413000355 | 1.338133854 | 0.504041204 |
| Nitab4.5_0005990g0040 | AT5G51990.1 | C-repeat-binding factor 4 | NA | NA | NA | NA |
| Nitab4.5_0005990g0050 | AT5G51990.1 | C-repeat-binding factor 4 | -5.727083194 | 0.075794775 | 0.573517412 | 0.726295127 |
| Nitab4.5_0006565g0010 | AT4G25470.1 | C-repeat/DRE binding factor 2 | -0.851629246 | 0.759816898 | 0.517562758 | 0.769318223 |
| Nitab4.5_0025012g0010 | AT4G25490.1 | C-repeat/DRE binding factor 1 | NA | NA | NA | NA |

1 Tobacco Gene ID refers to Gene IDs in reference genome of Nicotiana_tabacum cv. K326 (Nitab-v4.5_genome_Scf_Edwards2017.fasta downloaded from the database of Solanaceae Genomics Network).

2 Top Hit Accession and Top Hit Annotation refers to the best BLAST hit by search through the Araport11 proteins database downloaded from The Arabidopsis Information Resource (TAIR).

3 log_2_FC means the log_2_ fold changes of RPKM values detected from the RNA-seq results of SgRVE6-OE3 and SgRVE6-OE5 compared with wild-type tobacco.

4 padj means the adjusted p-values calculated by DESeq2
